# Goal setting improves retention in youth mental health: a cross-sectional analysis

**DOI:** 10.1186/s13034-019-0288-x

**Published:** 2019-07-09

**Authors:** Alice J. Cairns, David J. Kavanagh, Frances Dark, Steven M. McPhail

**Affiliations:** 1 Centre for Rural and Remote Health, James Cook University, PO Box 341, Weipa, QLD 4874 Australia; 2 Centre for Children’s Health Research, School of Psychology and Counselling and Institute of Health and Biomedical Innovation, Queensland University of Technology, Brisbane, Australia; 3 Rehabilitation Academic Clinical Unit, Metro South Addiction and Mental Health Services, Metro South Health, Brisbane, Australia; 4 School of Medicine, University of Queensland, Brisbane, Australia; 5 Australian Centre for Health Services Innovation, School of Public Health and Social Work and Institute of Health and Biomedical Innovation, Queensland University of Technology, Brisbane, Australia; 6 Centre for Functioning and Health Research, Metro South Health, Brisbane, Australia

**Keywords:** Youth mental health, Goal setting, Retention, Disengagement, SMART

## Abstract

**Background:**

This study explored if a youth-specific mental health service routinely set goals with young people during initial intake/assessment and if goal setting and goal quality in this service was associated with patient retention.

**Methods:**

Consecutive initial assessments (n = 283) and administrative service data from two youth-specific health services in Australia were audited for evidence of goal setting, content and quality of the goal and number of therapy services provided after the intake/assessment process. Logistic regression was used to determine if goal setting was associated with disengagement after the assessment session, controlling for drug use, unemployment, age, gender, mental health diagnosis and service site. A consecutive sub-sample of 166 goals (74 participants), was analysed for goal quality. Each goal was assessed against three components of the SMART (specific, measurable, acceptable/achievable, realistic and timed goals) criteria; specific, measurable and timed; and assigned a goal quality score 1–3. A multiple regression explored whether goal quality was predictive of the number of sessions attended, controlling for the same variables as the logistic regression.

**Results:**

Goal setting was evident in the records of 187 participants (66%). Although most goals were for emotional management, 24% addressed improvements in function. Of the 166 goals analysed in depth, 95 were specific, 23 measurable, but none were timed. Not setting goals during initial assessments correlated with service disengagement (OR 0.30, p > 0.001). Goal setting was positively associated with more therapy sessions attended, regardless of goal quality rating.

**Conclusions:**

Engagement and retention of young people within mental health services can be challenging. Clinical tools such as goal setting may keep young people engaged in services longer, potentially improving clinical outcomes. Further research exploring the effectiveness of current youth service models on client-specific goal based outcomes is recommended.

## Introduction

Having a goal and writing it down are two important tasks anyone can do to improve the likelihood of achieving a desired outcome. Goal setting is regularly used by mental health and rehabilitation professionals to focus service provision on functional outcomes that are meaningful to the consumer [1, 2]. Goal setting can also support recovery through individualisation of outcomes [3].

Goal setting might be especially relevant for young people accessing youth mental health services. This group experiences high rates of distress, disability and restricted social participation, as evidenced by their high rates (19–33%) of not being in employment, education or training compared with 14% of the general population of 20–24 year olds [4–6]. Meaningful change in social participation, rather than just psychological symptom relief, is a key aim of youth-specific mental health services [7–9]. The extent these services are achieving this aim is unclear [10].

Patient-specific outcomes like goal-based outcomes may offer a clinician and youth friendly solution to this problem [11]. Although goal setting is common practice in delivering psychological therapies to youth [2], the influence of goal setting on motivation and clinical outcomes within this population have not been well established [12]. In other fields, goal quality does appear to have an impact on immediate performance of tasks aimed at achieving that goal. In cerebrovascular rehabilitation settings, patients with functional, measurable goals at service entry tend to have higher discharge scores on functional measures than ones who made general goal statements [13]; and specific, challenging goals improved immediate performance in cognitive and motor tasks [14]. In non-clinical settings, specific and challenging goals have been associated with greater effort and persistence from goal setters in comparison to vague or ‘easy’ goals [15]. This demonstrates the potential influence on specific tasks necessary for goal achievement. However, there is no clear evidence that goal setting influences retention of patients within a service. This is a particularly pertinent issue in youth mental health, where attrition before treatment completion is common [16].

This investigation explored the routine use of goal setting with young people experiencing mental health issues during the first use of a youth-specific mental health service. This study explored whether the occurrence and quality of goal setting are associated with subsequent patient retention. This aim of this investigation was to:

- identify if goal setting was occurring during the initial intake and assessment process and what demographic variables may be associated with goals being set;
- explore the quality of the goals being set and pilot a quality index score and;
- identify if the presence or quality of goals was associated with the level of patient retention.

## Methods

### Design, participants and ethical approval

This cross-sectional investigation audited 283 consecutive clinical charts from young people aged 12–25 years old accessing a non-government youth mental health service (*headspace*) in 2016. Ethical approval was granted by the Queensland University of Technology (Approval Number 1400000066).

### Setting

Two *headspace* centres in South East Queensland, Australia participated in this study. *headspace* is an Australian-wide initiative with over 100 centres spread throughout the continent. *headspace* provides services to 12–25 year olds with the primary aim of promoting and supporting early intervention for mental health issues as well as general health, vocational and substance use problems [7]. Referrals are received from young people themselves (self-referral), parents/guardians, general practitioners and other health professionals, tertiary government mental health services, schools or community based organisations, and family or youth courts. *headspace*, clinicians will refer to tertiary government mental health services if the mental health needs of the young person are specialised or the person is at immediate risk to themselves or others. Young people seeking help from a *headspace* centre have at least one initial intake and assessment session to determine the individual’s needs and suitability for the service. If considered appropriate after the initial assessment, they are referred to a *headspace* therapist to provide ongoing mental (or physical) health services [17]. Young people can be involved with other clinical or vocational programs while engaged with *headspace. headspace*, has a ‘no wrong door’ policy meaning young people can present or be referred for any issue without having to negotiate complex inclusion/exclusion service criteria [18].

### Procedure

Initial intake, assessment and administrative service data from consecutive charts were audited by one member of the research team with support from a second member to check and clarify any ambiguous data. Support from a *headspace* clinician at each site was also available to clarify any ambiguous clinical notes. Basic demographic and clinical data including age (in years); gender (*M*/*F*); self reported current or previous drug use (*yes*/*no*); documented mental health diagnosis (*yes*/*no*); whether the participant was employed or studying (*yes*/*no*), were collected from the participants’ clinical intake and assessment information. Administrative data for each participant included the total number of therapy sessions attended after the initial intake/assessment process (patient retention) and the *headspace* site the participant sought help from.

#### Service disengagement

If no therapy sessions were attended after the initial assessment, this was classified as service disengagement (coded *yes*/*no*). This portion of the sample was of particular interest to the research team. Patient charts were scanned for a stated reason for not continuing with the service.

#### Goal setting

During intake and assessment sessions, service intake clinicians are expected to elicit what the young person hopes to achieve by attending the service (goals). To identify if goal setting occurred, all intake and assessment clinician notes were reviewed by a health professional independent of the clinical team. Goals for therapy or service engagement were typically documented at the end of the clinical assessment document; however, the entire assessment notes were audited to ensure goals recorded elsewhere were not missed. The presence of goal setting was recorded as a dichotomous variable (*yes*/*no*).

#### Goal content and quality

The content of a sub-sample of 74 consecutive charts with a documented goal was examined. Goals from these charts were recorded verbatim for assessment of content and quality. Goal content was coded into pre-specified categories derived from previously reported reasons for help-seeking and functional concerns [5, 19]. Goals were allocated to one category only. Potential categories were*: Emotional management*, *relationship/interpersonal*, *vocational* (school/work), *living skills* (e.g. housing, life planning), *alcohol/drug* related and *physical health* (including sexual health). An ‘other’ category was included for goals that did not fit into any of the above categories. If a goal could plausibly be linked to more than one category, it was allocated to the category that corresponded to the intended outcome. For example, one participant’s goal was to ‘manage social anxiety to stay employed’. This goal would potentially fit both in the *emotional management* and *vocational* categories. Because the participant identified the intended outcome was to remain employed, the goal was allocated to the ‘vocational’ category.

*Goal quality* was determined by analysing each goal against the SMART (specific, measurable, achievable, realistic/relevant and timed) framework for goal setting [20]. Because of the complexity and personal nature of determining if a goal was realistic or achievable (which the investigators did not believe could be judged from the information available), those components were not included in the analysis. Therefore, goals were assessed by a *yes*/*no* outcome on being:

- Specific—did they define exactly what is being pursued?
- Measurable—was there a clear way to track completion?
- Timed—is there any reference to time frame?

Goal quality analysis was conducted by the first author and was reviewed by another member of the research team for accuracy. A third member of the research team was available to arbitrate disagreements, but this was not required.

To predict the influence of goals and goal quality on the sum of sessions attended, goals were allocated a quality index score, piloted in this study. This scores were: 0 (no goals recorded), 1 (goals were reported but did not adhere to any SMART category), 2 (at least one goal set per participant was specific), 3 (at least one goal set was specific and measurable), and 4 (at least one goal set was specific, measurable and timed).

### Analysis

To explore potential variables associated with the presence of goals during the initial assessment, univariate logistic regressions were used to explore if the presence of goal setting (dichotomous outcome variable) was associated with age, gender, work/study status, history of drug use, mental health diagnosis, service disengagement or the service site. Service disengagement data were not available for nine participants, because the reason for disengagement was outside of the control of staff or participants. Reasons included unsuitability for the service and referral elsewhere (e.g. to a tertiary mental health service; n = 5); moving outside of the service catchment areas (n = 3); not being an Australia citizen and therefore being ineligible to access services through the primary service delivery model (n = 1). Explanatory variables with p < 0.2 in univariate analyses were carried forward for inclusion in a multivariable logistic regression to identify variables associated with goal setting when effects of other potential predictors were controlled.

Due to the distribution of the outcome variable (sessions attended), a negative binomial regression model was used to examine if number of sessions attended was predicted by goal quality. To determine if the quality of goals predicted the number of sessions attended (retention), results from the goal quality analysis (n = 74) were used. Participants with no recorded goals were included as the referent group to which participants in goal score categories 1, 2, 3 or 4 (described above) were compared. Univariate analyses were conducted to examine whether potential co-variates (age, gender, work/study status, history of drug use, mental health diagnosis and service site) were also associated with number of sessions attended and those with p < 0.2 were carried forward for inclusion in the multivariable negative binominal regression. Analyses were conducted using Stata 13 [21].

## Results

### Participant characteristics and service data

The mean and median age of the sample was 18 years (SD = 3.1). There were more female participants than male (female = 167; 59%), more than a quarter of participants were not working or studying (n = 82; 29%), a mental health diagnosis was recorded for 101 (36%) participants and 129 (46%) reported current or previous drug use. There were 8% more participants recruited from one of the service sites (Site 1 = 153; 54%) in comparison to the other site. From 283 reviewed patient charts, at least one goal was recorded for 187 (66%) participants. The median (IQR) number of sessions attended excluding the intake/assessment sessions was 5 [2–10] and 55 (19%) participants disengaged from the service after the assessment session.

### Associations with goal setting

Univariate analyses examining factors associated with goal setting identified age, drug use, service site and disengagement to be carried forward for multivariable analyses (Table 1). When entered into a multivariable logistic regression, service disengagement and site were statistically significant at p < 0.01 (Table 1). Compared with the univariate analysis, there was very little change in the odds ratio, confidence interval or p-value for service site or disengagement in the multivariable model.

**Table 1:** Results from univariate and multivariate logistic regression n = 274, examining potential correlates of goal setting (dependent variable)

	Univariate			Multivariate^†^		
	OR	95% CI		OR	95% CI	
		Lower	Upper		Lower	Upper
Age	1.09*	1.00	1.18	1.07	0.98	1.69
Male	0.74	0.45	1.22	–	–	–
Not working or studying	0.73	0.43	1.24	–	–	–
Mental health diagnosis	1.02	0.61	1.70	–	–	–
Drug use	1.54	0.90	2.51	1.49	0.85	2.60
Service disengagement	0.30**	0.16	0.58	0.30**	0.15	0.59
Service site	2.06*	1.24	3.43	2.05*	1.19	3.53

*OR* odds ratio

*p ≤ 0.05; **p ≤ 0.001

^†^The overall model gave LR X^2^ (4) = 25.65, p < 0.001

### Association between goal quality and patient retention

**Fig. 1:**
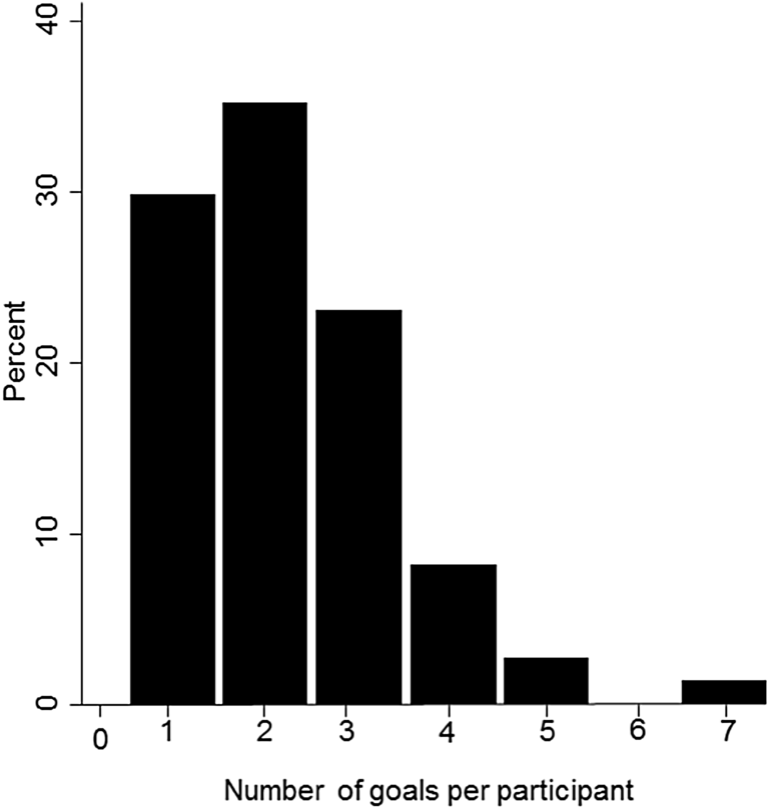
Number of goals recorded per participant (n = 74)

Among the 74 participants included in the sub-analysis of goal quality, 166 goals were analyzed, with 88% (n = 65) of participants reporting between 1 and 3 goals (Fig. 1). The frequency of goal categories has been described in Table 2. Goals to improve emotional management and well-being were the most frequently recorded, with support for depression and anxiety symptoms contributing to half of these. Goals in the ‘other’ category were: stay out of jail (n = 1), engage with psychologist/talk to someone (n = 4), be a better person (n = 1), get a handle on life (n = 1), be normal (n = 1) and increase my mental health to increase functioning (n = 1). That final goal was allocated to the ‘other’ category, as the authors were unable to specify what aspect of the participant’s mental health or area of functioning was the focus.

**Table 2:** Type and frequency of goals reported by 74 help-seeking young people: 166 goals analysed

Goal category	N (%)
Emotional management/feelings	107 (64%)
Depression/mood symptoms	26
Anxiety	25
Self esteem	12
Stress management	11
General coping	10
Anger management	7
Suicide/self-harm	6
Eating disorder	3
Psychotic symptoms	3
Trauma counselling	2
Motivation	2
Relationship/interpersonal	20 (12%)
Vocational (work/study)	11 (7%)
Living skills (e.g. housing, community access)	9 (5%)
Alcohol and drug	6 (4%)
Physical health	3 (2%)
Other	9 (5%)

None of the analyzed goals met full criteria for being specific, measurable and timed, so none scored 4 on the quality index. Ninety-five goals (57%) were identified as being specific and 23 were measurable (14%). All goals that were considered measurable were also specific. None of the goals included a timeframe. Of the 23 measurable goals, 22 were identified as measurable as they inferred a dichotomous yes/no measure (e.g. “*stop smoking cannabis*” or “*get a job*”).

Results from the negative binomial regression indicated that the presence of a goal compared with no goal was associated with more sessions attended (Table 3). The multivariable regression identified that no history of drug use was associated with a higher number of sessions attended. History of drug use reached significance, p < 0.05, in the multivariable analysis likely as a result of the interaction with gender and the increased effect on the dependent variable (number of sessions attended). It was interesting to note that incident rate ratio estimates for the association between goal quality categories and number of sessions attended were quite consistent across the three goal quality categories indicating that goals that were specific, or specific and measurable did not tend to give superior patient retention than ones that did not meet these criteria.

**Table 3:** Results from univariate and multivariate negative binominal regressions examining potential correlates of number of sessions attended (dependent variable) n = 166

	Univariate			Multivariable^†^		
	IRR	95% CI		IRR	95% CI	
		Lower	Upper		Lower	Upper
Goal quality^a^						
Not specific or measureable	2.72**	1.54	4.80	2.76**	1.57	4.87
Specific goal not measureable	2.44**	1.59	3.75	2.48**	1.60	3.84
Specific and measurable goal	2.33*	1.33	4.08	2.30*	1.30	4.07
Age	1.04	0.96	1.12	n/a	–	–
Male	0.67*	0.46	0.97	0.76	0.54	1.08
Not working or studying	0.93	0.63	1.38	n/a	–	–
Mental health diagnosis	1.24	0.85	1.81	n/a	–	–
History of drug use	0.75	0.52	1.09	0.68*	0.48	0.97
Service site	1.56*	1.07	2.25	1.16	0.81	1.67

*IRR* incident rate ratio

*p ≤ 0.05; **p ≤ 0.001

^†^Overall model LR X^2^ (6) = 33.24, p < 0.001

^a^No goal is the referent comparison category

## Discussion

More than two-thirds of young people in this study set goals during their initial engagement and assessment sessions with a youth mental health service. Of the 74 participants included in the sub-analysis of goals, 52 (30%) identified more than one goal. This is congruent with previous research from youth mental health services where the majority of young people report more than one reason for help-seeking [22] and young people find goal setting to be acceptable and valued [12].

### Factors associated with goal setting

In this sample, goal setting was not significantly associated with age, gender, presence of mental health diagnosis, history of drug use or vocational functioning. These results are encouraging, as they indirectly suggest the likely acceptability of goal setting amongst a broad range of young people. Not setting a goal was correlated with an increased likelihood of a young person not returning to the service for ongoing therapy (service disengagement). This result was evidenced in both the association between goal presence and disengagement (Table 1), and between goal quality and number of therapy sessions attended (Table 3). The mechanisms underpinning this result are worth further exploration. It is possible that those that disengaged from the service after the assessment session did not set a goal, as it was their intention not to return. However, this moment of discussing goals during the assessment may provide an opportunity for a clinician to change a young person’s perspective of the service. That person after all, has made the effort to attend the service for the intake assessment presumably indicating that they are likely to have an objective in mind that could plausibly be articulated as a goal.

There is very little information about disengagement from youth early intervention services comparable to *headspace*, and the authors could find no other studies examining the influence of goal setting on disengagement. Comprehensive school-based engagement models postulate goal setting, focused on task rather than ability, as important for school engagement, but until the present study it was unknown if this would also apply to health services [23, 24]. Further research exploring the motivation to attend ongoing intervention pre and post assessment may give insight into the potential mediating role of goal setting. It is possible that strengthening goal setting practices could reduce the rate of service disengagement. In this study, just having a goal significantly predicted an increase in the number of sessions attended, although there was not a clear association between the quality of goals and the number of sessions. Furthermore, increased sessions may not necessarily be a positive outcome if the purpose of intervention were unclear, or the purpose of the intervention was rapidly achieved.

The influence of site on goal setting suggested a possible disparity between sites in the implementation of routine goal setting and recording during the initial assessment. It is possible that the site differences were due to differing staffing competencies/characteristics or service cultures, or to participant characteristics such as the extent their initial motivation to attend the service was related to a consciously articulated goal [25, 26]. The influence of site was not significantly correlated with patient retention once other covariates were included in the analysis (Table 3). This indicates that any characteristics that may relate to site differences did not significantly influence patient retention. Lastly a history of drug use was associated with a reduced number of therapy sessions attended. This is congruent with previous literature exploring mental health service disengagement [27]. The underlying reasons for this could not be explored in this study but is an area of research requiring further attention.

### SMART goals and content

Results on the content focus of goals were consistent with national *headspace* data, that 71.6% of young people were having problems with feelings, 18.4% reported help-seeking for concerns with role functioning and 6.6% had physical health issues [5]. Similar services outside of Australia, such as, *Jigsaw*, the Irish national youth early intervention service also report most young people present for issues relating to feelings such as anxiety and worry, anger and thoughts of hurting one’s self being most commonly reported [22]. Tangible outcomes have been postulated as being potentially more important to young people and their families [28] and it is likely that the intended outcome for some of the emotional goals was subsequent improvement in functioning, but that hypothesis could not be tested in the current study.

In this study, most goals did not adhere to the SMART criteria. Negotiating specific, realistic and measurable goals with service users is perceived to be time consuming [20], which may have constrained the extent that this could occur. Almost all of the measurable goals used a dichotomous measurement, and while such outcomes are measurable, they do not allow for partial success. This may inadvertently be detrimental to individuals who do not achieve a positive result [29]. While the current study suggested that setting goals, regardless of quality, is more helpful than no goals, evaluation of the extent of goal attainment was outside the scope of this research, and specific, measurable and realistic goals may have resulted in superior outcomes.

Effective goal setting is challenging, but idiographic measures may provide an alternative evaluation tool to global assessments of functioning, more sensitive to outcomes meaningful to consumers [11, 30]. This study did not explore the process for reviewing goals. However, previous research reported young people could not always remember the goals they had set at entry into services and that they were not systematically reviewed [12]. The process for goal evaluation and feedback remains an important area for future research.

### Implications for practice

This study highlighted that although most young people in our sample are setting goals when they engage with youth services, few goals were specific and measurable. Regardless of the goal quality, any form of goal setting appeared to reduce the risk of patients disengaging immediately after assessment, and was related to more sessions being attended. Idiographic outcome measures, such as goal setting did not appear to be used to their full potential at these two sites, despite the desire from youth services to improve functional outcomes [31]. Introduction of tools such as the MyLifeTracker have significant potential in demonstrating meaningful change for young people [32]. Practitioners working in youth mental health services may find it beneficial to consider increasing the focus on goal setting to improve client retention and measurement of client-desired change to understand effectiveness of therapy [33].

### Limitations

Although the goals coded in this study were written in a manner that suggested they were identified by service users, the authors were unable to validate this as the data were retrospective and were collected from clinical charts. It is possible that the goals recorded were not always negotiated between the young person and the clinician but instead a statement by either the young person or clinician, and documented with or without agreement on the achievability of the goals. Future studies, reporting the quality of the goals setting process may identify whether the achievability of goals is associated with patient retention. The commitment of parents/guardians to support the young person to accesses treatment is also a likely factor in treatment retention however this was unable to be explored in this current study. This study focused on goal setting with young people at the intake and assessment phase of service engagement and did not examine the presence and content of goals set during ongoing therapy. Some SMART goals could have been subsequently set by therapy staff. The process for setting goals, goal feedback and staff’s perceptions on the utility of setting goals were not explored in this study and could provide valuable information for service improvement in the future. Lastly, a dichotomous assessment of engagement in work or study was a basic determination of occupational functioning and does not provide any assessment of the quality of engagement or the supports an individual might be receiving. It is possible that a more detailed assessment of the quality of vocational functioning might identify an association between goal setting and concurrent function. It is also important to note that no assessment of later functioning or other outcomes was included in this study, and that may have provided additional insight into the role of goal setting and goal quality.

## Conclusion

This study successfully assessed the rates and quality of goal setting during initial engagement at youth health services and explored the associations between goal setting and patient retention. Clinicians working in this field and particularly intake/assessment staff in youth-specific mental health service should consider the role of goal setting at the initial phase of patient engagement. This study has highlighted that the majority of young people were setting goals, but those goals were not always specific, rarely measurable, and when dichotomous, they were not conducive to indicating satisfaction with partial achievement. Further research is needed to understand the mechanism of goal setting in improving patient retention, with the ultimate aim of improving meaningful patient-specific outcomes.

## Data Availability

The datasets used and analysed during the current study are available from the corresponding author on reasonable request.

## Abbreviations

SMART: specific, measurable, acceptable/achievable, realistic and timed (goals)
